# Unveiling the Antioxidant Therapeutic Functionality of Sustainable Olive Pomace Active Ingredients

**DOI:** 10.3390/antiox11050828

**Published:** 2022-04-24

**Authors:** Javier Quero, Lina F. Ballesteros, Pedro Ferreira-Santos, Gustavo R. Velderrain-Rodriguez, Cristina M. R. Rocha, Ricardo N. Pereira, José A. Teixeira, Olga Martin-Belloso, Jesús Osada, María Jesús Rodríguez-Yoldi

**Affiliations:** 1 Department of Pharmacology and Physiology, Forensic and Legal Medicine Veterinary Faculty, University of Zaragoza, 50013 Zaragoza, Spain; javierquero94@gmail.com; 2 CEB-Centre of Biological Engineering, Campus de Gualtar, University of Minho, 4710-057 Braga, Portugal; linafernanda37@ceb.uminho.pt (L.F.B.); pedrosantos@ceb.uminho.pt (P.F.-S.); cmrocha@ceb.uminho.pt (C.M.R.R.); rpereira@deb.uminho.pt (R.N.P.); jateixeira@deb.uminho.pt (J.A.T.); 3 LABBELS–Associate Laboratory, 4710-057 Braga, Portugal; 4 Alianza Latinoamericana de Nutricion Responsable Inc., 400 E Randolph St Suite 2305, Chicago, IL 60611, USA; coordinacion.cientifica@alanurla.org; 5 Department of Food Technology, University of Lleida-Agrotecnio Center, Av. Alcalde Rovira Roure 191, 25198 Lleida, Spain; olga.martin@udl.cat; 6 Department of Biochemistry and Molecular and Cell Biology, Veterinary Faculty, University of Zaragoza, 50013 Zaragoza, Spain; josada@unizar.es; 7 CIBERobn, ISCIII, 28029 Madrtid, Spain; 8 IIS Aragón, IA2, 50013 Zaragoza, Spain

**Keywords:** olive pomace waste, ohmic heating, apoptosis, Caco-2 cells, ROS

## Abstract

Olive pomace (OP) is the main residue that results from olive oil production. OP is rich in bioactive compounds, including polyphenols, so its use in the treatments of diseases related to oxidative stress, such as cancer, could be considered. The present work aimed to study the biological properties of different OP extracts, obtained by ohmic heating-assisted extraction and conventional heating, using water and 50% ethanol, in the treatment and prevention of colorectal cancer through Caco-2 cell models. Additionally, an in-silico analysis was performed to identify the phenolic intestinal absorption and Caco-2 permeability. The extracts were chemically characterized, and it was found that the Ohmic-hydroethanolic (OH-EtOH) extract had the highest antiproliferative effect, probably due to its higher content of phenolic compounds. The OH-EtOH induced potential modifications in the mitochondrial membrane and led to apoptosis by cell cycle arrest in the G1/S phases with activation of p53 and caspase 3 proteins. In addition, this extract protected the intestine against oxidative stress (ROS) caused by H_2_O_2_. Therefore, the bioactive compounds present in OP and recovered by applying a green technology such as ohmic-heating, show promising potential to be used in food, nutraceutical, and biomedical applications, reducing this waste and facilitating the circular economy.

## 1. Introduction

Extra-virgin olive oil (EVOO), mechanically extracted from the olive fruit is one of the most representative foods in the traditional Mediterranean diet (MD) and is considered a key element associated with several health-beneficial effects. Epidemiological studies show a direct relationship between olive oil consumption and a decrease in the occurrence of different types of cancer, cardiovascular risk factors, age-related processes, chronic inflammatory disorders, and inflammatory bowel diseases [1]. These beneficial effects are mainly attributed to the phenolic compounds present in virgin olive oil [1]. The production of olive oil is very important in Mediterranean countries such as Spain, Portugal, and Italy. On the other hand, its production generates a large amount of waste [2], with olive pomace (OP) as one of the main fractions [3]. Industrial olive processing wastes are a problem because they harm the environment since their high organic acid concentration turns them into phytotoxic materials. However, they also contain valuable nutritional substances [4].

The malaxation step and crushing the olive, during the obtaining of virgin olive oil, are the main steps to obtain the paste for separation of the oil. Subsequently, the separation of the oil phase is produced through centrifugation or pressure. In Mediterranean countries, a three-phase centrifugation system is used [5]. In this way, the phases obtained are: the pomace (solid), the olive oil, and the wastewater [6,7]. These three phases are rich sources of polyphenols with a large spectrum of biological activities [8]. After milling, only 2% of the phenolic compounds in olives is transferred to the oil and as much as 98% is retained in the by-products. In particular, OP has a high concentration of minerals, sugars, and phenolic compounds as tyrosol and hydroxytyrosol (HT) [7]. Therefore, these valuable compounds could be obtained from the olive oil industrial residues, and used in the development of functional food ingredients and nutraceuticals.

In addition, there is evidence that phenolic compounds extracted from the *Olea europaea* L. tree and by-products obtained from olive processing, can be used as natural antioxidants and antimicrobial additives to improve the conservation and nutritional properties of food products [9], as well as some potential biomedical applications [10]. Phenolic compounds have been shown to have significant anticancer effects in vivo and in vitro in different types of human cells without affecting healthy cells [11].

Currently, available cancer therapies are associated with a range of side effects that reduce the overall quality of life. Therefore, it is essential to identify alternative pharmaceuticals with the aim of being more effective and less toxic. Natural products, including phenolic compounds, could be a great alternative therapeutic agent. In this way, olive oil by-product extracts from *Olea europaea* L. and enriched with HT produced decreased cell viability in HCT8-β 8 human colon cancer cells [12]. Furthermore, the flavonoid quercetin (QUE), found in waste olive products, exhibits an anticancer effect against different types of human cancers such as breast, colorectal, stomach, head and neck, lung, ovarian, melanoma, and leukemia by different mechanisms of action [13,14,15].

At present, the methods for obtaining polyphenols from oleic by-products have been improved with new technologies with lower energy consumption and the elimination of additives [16]. Thus, ohmic heating (OH) demonstrates promising technology for assisting in an efficient extraction of biocompounds. OH is capable of promoting a fast, homogeneous, and selective extraction by the application of moderate electric fields through raw material, causing disruption of cell membrane resulting in the formation of pores (electropermeabilization) or cell disintegration, allowing the diffusion of intracellular components [17,18,19]. Furthermore, this new “green” technology may allow a reduction in the environmental impacts caused by the traditional extraction processes, decreasing the water/solvent use, processing times, waste generation, and energy consumption. This represents a hallmark in a circular economy by obtaining new high-value ingredients for health-related products (nutraceuticals and pharmaceuticals). Thus, the main objective of this research work was to obtain an extract rich in phenolic compounds through conventional and/or OH using safe solvents. The OP extracts were chemically characterized in terms of phenolic content and antioxidant activity, and the potential therapeutic was evaluated in Caco-2 cells. Furthermore, the theoretical absorption percentage of individual phenolic compounds was assessed by bioinformatic tools. In this paper, we focused our attention on the secondary metabolites contained in OP by-product materials derived from olive oil production and their ability to reduce oxidative stress-related diseases such as colon cancer. This disease is caused by an uncontrolled proliferation of normal cells that become malignant cells due to genetic or epigenetic changes [20]. This study is part of a project that we have been developing in our laboratory for a few years on the prevention and treatment of colon cancer with plant extracts and organometallic complexes.

As a final point, it is important to highlight that in vitro experiments represent the first step in evaluating the health effect of new functional ingredients. Although further clinical studies are needed, the results shown here clearly indicate the anticancer effect of this residue and pave the way for the exploitation of olive pomace residues as a functional ingredient with biomedical applications.

## 2. Materials and Methods

### 2.1. Raw Materials and Chemicals

OP waste was supplied by Indulleida S.A. (Alguaire, Lleida, Spain). As soon as it was received, OP was placed in an oven and dried for 48 h at 60 °C. Subsequently, it was milled and sieved to obtain a material with a particle size between 0.45 to 0.9 mm and 7.41 ± 0.08% (*w*/*w*) of moisture determined through a moisture analyzer MAC 50/1/NH (Radwag, Radom, Poland). Finally, OP was kept at room temperature in a dark bottle until evaluated. All the analytical grade chemicals were acquired from Fisher Scientific (Leicestershire, UK) and Sigma-Aldrich (Steinheim, Germany).

### 2.2. Determination of the OP Waste Proximate Composition

The raw material was characterized in terms of total carbohydrates, lignin, protein, ashes, lipids, and minerals. Prior to cellulose, hemicellulose and lignin determination, the extractives were removed from OP waste in a SoxtecTM 8000 extraction system (Foss, Hilleroed, Denmark) using distilled water in the first stage, followed by ethanol 95% (*v*/*v*) [21]. The extractive free OP samples were dried at 60 °C until constant weight and then, cellulose, hemicellulose and lignin contents were determined, submitting the samples to a two-step sequential acid hydrolysis according to [22]. Structural carbohydrates in the resulting solutions were identified and quantified by high-performance liquid chromatography (HPLC) using a Refractive Index detector and an 87 H column (300 × 7.8 mm, Aminex, BioRad) at 60 °C, eluted with 0.005 M H_2_SO_4_ at a flow rate of 0.6 mL/min. The contents of cellulose (as glucose) and hemicellulose (as xylose and arabinose), as well as the acetyl groups, were calculated from HPLC data. The Klason lignin (insoluble lignin) present in OP was gravimetrically quantified from the insoluble solid residue. Additionally, the soluble lignin content was calculated as described by Mussatto and Roberto [23].

Fat quantity present in OP waste was determined in a SoxtecTM 8000 extraction system (Foss, Hilleroed, Denmark), utilizing petroleum ether as a solvent for 1 h, according to Ballesteros et al. [24]. Ashes were determined by incinerating the samples at 550 °C overnight. Nitrogen content was carried out through the Kjeldahl method by using a TecatorTM digestion unit (Foss, Hilleroed, Denmark) and a KjeltecTM 8400 analyzer (Foss, Hilleroed, Denmark), where the protein quantity was calculated by utilizing the N_2_ × 6.25 conversion factor.

The mineral content quantification was performed by inductively coupled plasma optical emission spectroscopy (ICP-OES) after digestion of 0.5 g of OP with a mixture of 9 mL HNO_3_ 65% (*v*/*v*) and 1 mL H_2_O_2_ 35% (*v*/*v*) using a microwave digestion system (Speedwave^®^ four V.8.0 Berghof, Harretstrasse, Germany). The measurements were carried out in an Optima 8000 ICP-OES (Perkin Elmer, MA, USA), operating with axial plasma view and 1400 W at different wavelengths. A calibration curve was prepared from ICP standards to quantify the minerals present in the samples.

### 2.3. Extraction of Bioactive Compounds from Olive Pomace Wastes

#### 2.3.1. Ohmic Heating (OH)

Extractions using the OH technique were carried blending 2 g of OP with 20 mL of solvent (ultrapure water or ethanol 50% (*v*/*v*)). Experiments were performed into a double-jacketed cylindrical glass reactor containing stainless-steel electrodes in both edges [17]. The distance between electrodes was kept constant (3.5 cm) and the samples were slightly agitated in order to ensure their uniformity. The conductivity of the samples was enhanced by adding a 70 mM NaCl solution to the solvents, achieving values of 7.15 mS/cm and 2.63 mS/cm for the ultrapure water and ethanol 50% (*v*/*v*), respectively. The temperature was controlled by adjusting the voltage output of a function generator (Agilent 33.220 A, 1 Hz–25 MHz, and 1–10 V, Penang, Malaysia) with a sinusoidal wave at a frequency of 25 kHz, linked to an amplification system (Peavey CS3000, 0.3–170 V, Meridian, MS, USA). The electric field was approximately 4 V/cm, and the temperature (83 ± 2 °C) was monitored by a k-type thermocouple (temperature ± 1 °C, Omega Engineering, Inc., Stamford, CT, USA), which was put in the geometric center of the sample and linked to data logger software (USB-9161, National Instruments Corporation, Austin, TX, USA). After 30 min of heating, the samples were recollected and rapidly cooled down in an ice bath during 15 min to break the extraction. Later, they were centrifuged (2500 g, 20 min), filtered through 0.22 µm membranes, and kept at −20 °C or lyophilized for the respective tests.

#### 2.3.2. Conventional Heating (CH)

CH experiments were performed maintaining the solid-liquid ratio, solvents, time, and temperature as described in the OH experiments. For the extractions, the mixtures were placed into 100-mL Shots duly covered to avoid solvent loss and then heated in a shaking water bath (Julabo SW22, Seelbach, Germany) at 170 rpm. Finally, the extracts were treated and stored as those obtained with the OH technique.

### 2.4. Analytical Methodology for Extracts Characterization

#### 2.4.1. Extraction Yield

The extraction yield was carried out by gravimetric analysis. In brief, 1 mL of each extract was weighed before and after being placed at 100 °C overnight. The results were expressed as g recovered extract per 100 g material (% (*w*/*w*)).

#### 2.4.2. Total Phenolic Compounds

The total content of phenolic compounds (TPC) was carried out using the Folin-Ciocalteu method described previously by Ballesteros et al. [25]. A calibration curve was prepared using gallic acid as standard (200–2000 mg/L). The TPC was expressed as milligram gallic acid equivalent per gram of dry material (mg GAE/g OP).

#### 2.4.3. Individual Phenolic Compounds Determination

The phenolic compounds present in the extracts obtained from OP waste were analyzed by ultra-high-pressure liquid chromatography (UHPLC) according to Jesus et al. [26]. Briefly, a Shimadzu Nexpera X2 UHPLC chromatograph equipped with a Shimadzu SPD-M20 A diode array detector and a reversed-phase Aquity UPLC BEH C18 column by Waters (2.1 mm × 100 mm, 1.7 µm particle size) at 40 °C were used to separate and identify the compounds. The eluent involved an aqueous 0.1% formic acid solution as the solvent A, and acetonitrile as the solvent B under the following gradient profile: 95% A and 5% B (0 to 5.5 min), a linear increase to 60% B (5.5 to 17 min), a linear increase to 100% B (17 to 18.5 min) and finally, 95% A and 5% B (18.5 to 30.0 min) for the column equilibration. The flow rate of the mobile phase and the injection volume of the samples were maintained to 0.4 mL/min and 1 µL, respectively. Different HPLC grade phenolic compounds were used to prepare standard curves to identify and quantify the compounds found in the extracts. The responses of the UV detector were integrated using the LabSolutions software (Shimadzu, Kyoto, Japan).

#### 2.4.4. Antioxidant Activity

The antioxidant activity of OP extracts was performed by two different procedures including 2,2-diphenyl-1-picrylhydrazyl (DPPH) radical scavenging activity assay [25], and ferric reducing antioxidant power (FRAP) assay [27] according to Ballesteros et al. DPPH percent inhibition data were plotted as a function of Trolox concentration (used as standard) to find inhibition concentration at 50% (IC50), and then, the values were ex-pressed as micromoles of Trolox equivalent per gram of dry weight material (μmol TE/g OP). On the other hand, the FRAP assay used a calibration curve made from an aqueous solution of ferrous sulphate (200–1000 μM), and the results were expressed as micromoles of ferrous equivalent per g of dry weight material (μmol Fe(II)/g OP).

#### 2.4.5. Structural Characterization

The chemical groups and bonding arrangement of constituents found in the OP waste and OP extracts were performed by Fourier transform infrared spectroscopy (FTIR). Thus, a Bruker Alpha II spectrometer (Ettlingen, Germany) with a diamond crystal attenuated total internal reflectance (ATR) accessory was used. The tests were registered from 4000 to 400 cm^−1^ wavenumber range, scanning 24 times each sample at a resolution of 2 cm^−1^, and operating Opus software.

### 2.5. Theoretical Absorption Percentage of Individual Phenolic Compounds

The theoretical absorption percentage of the individual phenolic compounds from OP was addressed by using the chemical structures and SMILES (simplified molecular-input line-entry system) codes, obtained from the PubChem Open Chemistry Database, of those compounds identified by UHPLC [28]. Moreover, the relevant molecular properties related to their intestinal absorption molecular weight (MW; g/mol), total polar surface area (TPSA), octanol/water partition coefficient (LogPo/w), Lipinski’s rule of five (LIRF), and theoretical percentage of absorption (% Abs) were further obtained by using the “Molinspiration online property calculation toolkit” [29] as described by Velderrain-Rodríguez et al. [30]. Lastly, the Caco-2 permeability was predicted using the online program pkCSM [31] and displayed as the logarithm of the apparent permeability coefficient (log Papp) expressed in 10^−6^ cm/s.

### 2.6. Cell Culture

Caco-2 human colon carcinoma cells (clone TC7) provided by Dr. Edith Brot-Laroche (Université Pierre et Marie Curie-Paris 6, UMR S 872, Les Cordeliers, France) were used. Cells were cultured in Dulbecco’s Eagle Medium (DMEM) (Gibco Invitrogen, Paisley, UK). Fetal bovine serum (FBS) (20%,) non-essential amino acids (1%), penicillin (1000 U/mL) (1%), Streptomycin (1000 μg/mL) (1%), and amphotericin (250 U/mL) (1%) were added to the medium. The cells grew in a humidified atmosphere of 5% CO_2_ at 37 °C, being trypsinized (0.25%−1 mM EDTA) and subsequently cultured at a density of 5 × 10^5^ cells/cm^2^ in 25 cm^2^ plastic flasks. The culture medium was changed every 2 days. Cell confluence (80%) was determined by optical microscopy and was reached 15 days after seeding, observing cells differentiated into enterocytes. At 24 h post-seeding, the cells were undifferentiated and were used for the treatment with the extracts.

### 2.7. Cell Treatment and Antiproliferative Property Analysis

For the cytotoxicity assays, OP extracts diluted in culture medium at a concentration of 1.5 mg/mL were used, and the cells were treated for 72 h. The antiproliferative effect was measured with the fluorometric cell viability resazurin assay [32] being expressed as a percentage with respect to the control. The IC_50_ value was calculated in all the conditions tested and represents the concentration of the extract capable of reducing the viability of the cells by half.

### 2.8. Measurements of Apoptosis

Once the cells were seeded in 25 cm^2^ flasks (5 × 10^5^ cells/cm^2^), they were treated with the extracts for 72 h at the previously obtained IC_50_ concentration. Subsequently, the methods described by Sánchez-de-Diego et al. [33] were used.

### 2.9. Propidium Iodide Stainning of DNA Content and Cell Cycle Analysis

The OP treated Caco-2 cells were fixed in 70% ice-cold ethanol and stored at 4 °C for 24 h. After centrifugation (2500 rpm, 5 min), cells were rehydrated in PBS and stained with propidium iodide (PI) solution (50 μg/mL) containing RNase A (100 μg/mL). PI stained cells were analyzed for DNA content in a PCSArray BD as described by Sánchez-de-Diego et al. [33].

### 2.10. Flow Cytometry Mitochondrial Membrane Potential Assay

Once the cells were seeded, they were treated for 72 h with medium with/without OP extracts. The determination was performed following the hexamethylindodicarbocyanine iodide (DiIC1) method previously described [33].

### 2.11. Determination of Caspase 3 and P53

Caco-2 cells were plated in a 25 cm^2^ flask at a density of 3 × 10^5^ cells per flask and incubated for 24 h under standard cell culture conditions. Then, 1.5 mg/mL olive pomace solution was added to a flask and incubated for 72 h. Likewise, the cells were collected and processed following the instructions of Sánchez-de-Diego et al. [33]. To finish, 50 µL of each sample was incubated with 5 µL of active anti-caspase-3 (BD Pharmigen, Clone C92-605) and 5µL p53 antibody (Miltenyi, Clone REA609). Fluorescence was measured by flow cytometry using a Beckman Coulter Gallios (Brea, CA, USA) equipped with a blue solid diode laser (488 nm) and a red solid diode laser (635 nm). Caspase-3 was determined at the excitation 488 nm wavelength and the emission at 525 nm. In the case of p53, the excitation at 635 nm and the emission at 660 nm were measured.

### 2.12. Determination of Intracellular Levels of Reactive Oxygen Species (ROS)

The cells were seeded in a 96-wells plate at a density of 4 × 10^3^ cells/well. The intracellular level of ROS was assessed using the dichlorofluorescein assay as previously described by Sánchez-de-Diego et al. [33]. Cells were cultured before oxidative stress induction and then incubated with OP for 24 h. After that, the medium was removed, cells were washed twice with phosphate-buffered saline, and incubated for 1 h with 20 μM 2′,7′–dichlorofluorescein diacetate (DCFH-DA) in PBS at 37 °C. The formation of the fluorescence oxidized derivative of DCF was monitored at an emission wavelength of 535 nm and an excitation of 485 nm in a multiplate reader. A measure at time “zero” was performed, cells were then incubated at 37 °C in the multiplate reader, and generation of fluorescence was measured after 20 min. ROS levels were expressed as a percentage of fluorescence compared to the control. The obtained values of fluorescence intensity are considered as a reflection of total intracellular ROS content.

### 2.13. Statistical Analysis

Data were presented as mean ± standard deviation (SD) for a minimum of three independent experiments. Data were compared using one-way analysis of variance (ANOVA) and significant differences at *p* < 0.05 were obtained using a Bonferroni’s Multiple Comparison Test. The statistical analysis was performed using the software GraphPad Prism (Version 5.02, San Diego, CA, USA).

## 3. Results and Discussion

### 3.1. Chemical and Mineral Composition of OP

The proximate composition of raw material OP is summarized in Table 1. The results show that the major elements of the OP composition are polysaccharides (35.1%), polymerized into hemicellulose and cellulose structures. The hemicellulose corresponds to an average of approximately 19% being xylose and arabinose monosaccharides the main components, while cellulose (as glucose) represents 16.2% of OP composition on a dry weight basis. On the order hand, lignin represents 31.7%, being 20.5% klason lignin and 11.2% acid-soluble lignin. The water and ethanol soluble extractives correspond to 25.2% of total OP waste and other constituents including the fat and protein content represent 12.1% and 5.7% of total OP composition, respectively.

The ashes (inorganic fraction) represent 4.6% of OP composition and the most relevant minerals include potassium (2798.4 mg/Kg), calcium (352.0 mg/Kg), and magnesium (145.4 mg/Kg). These findings are in agreement with those reported by Ribeiro et al. [34], who obtained similar values of proximate composition for crude OP (predominant olive varieties: Galega Vulgar) obtained from the Inner Centre Region of Portugal.

### 3.2. Extraction Yield, Phenolic Composition, and Cell-Free Antioxidant Activity of OP Extracts

In this work, a green, sustainable, and economical methodology was designed to enhance value to the OP by-product, which is currently generated in large quantities in the Mediterranean area, recovering its biomolecules into (bio)functional ingredients, such as phenolic compounds.

The influence of solvent (water or ethanol) and the extraction techniques (OH-assisted or CH) on the extraction yield, TPC, and cell-free antioxidant action by DPPH and FRAP assays are shown in Table 2. The results indicate that the extraction yield increased 7–15% when the extraction process was performed under electric fields effects (OH) when compared to the CH, as expected. However, the solvents used showed no influence on the extraction yield. As reported in the literature, the use of specific solvents is responsible for the selectivity of extraction and dissolution of intracellular biocompounds. For example, secondary metabolites with antioxidant capacity present in plants and by-products, such as phenolic compounds, show higher affinity/solubility in polar solvents due to the presence of a hydroxyl group [35]. Therefore, in this work, polar solvents such as water and ethanol were used as safe, and effective environmentally friendly solvents, making the process more sustainable [36].

On the other hand, the TPC values obtained for OP extracts by the different extraction methodologies are significantly influenced by the solvent but not by the extraction technique, with the highest TPC obtained in the hydroethanolic extracts (Table 2). The TPC evaluated by the Folin-Ciocalteu assay presents values between 12.08 and 17.67 mg of GAE/g OP. Ribeiro et al. [34] reported TPC values above 20 mg GAE/g OP using methanol as a solvent. On the other hand, Nunes et al. [37] quantified about 30 mg GAE/g OP using water as the extraction medium.

The OP extracts, rich in phenolic compounds, were used to evaluate the chemical radical scavenging capacity by DPPH assay, and chemical reduction power by FRAP assay centered on the reduction in an iron complex Fe^3+^ to Fe^2+^. As shown in Table 2, the DPPH results present similar values when comparing both extraction technologies used. However, the hydroethanolic extracts, regardless of the extraction method, have a higher capacity to scavenge free radicals, when compared with the aqueous extracts. The FRAP assay also shows higher antioxidant activity for hydroethanolic extracts compared to aqueous ones, with values approximately 46% and 38% higher for OH and CH methods, respectively.

These results are in agreement with the previous statement that ethanol has an important role in the recovery of biomolecules with higher antioxidant capacities [36]. Several authors reported that OP-derived extracts have an interesting antioxidant capacity that may be related to the content of phenolic compounds [34,37,38]. For example, Ribeiro et al. [34] evaluated the antioxidant capacity of OP extracts using three different methods (DPPH, ABTS, and ORAC), and their results showed high antioxidant activity for the methanolic extracts of OP. Nunes et al. [37] report a relationship of antioxidant activity (DPPH, ABTS, and FRAP) with HT content for water-soluble extractives of OP by-product of several olive varieties from the northeast region (Behind-the-hills) and south (Alentejo) of Portugal.

The obtained results for the antioxidant chemical tests are in agreement with the TPC and individual phenolic content (Table 2 and Table 3), and also showed that the phenolic compounds are probably the major antioxidant contributors of OP extracts.

A total of 22 phenolic compounds belonging to the groups of hydroxytyrosol and tyrosol derivatives, phenolic acids, flavonoids, and stilbenes were identified according to their corresponding standards and are shown in Table 3.

In general, the extraction with water/ethanol mixtures lead to the highest extraction of almost all phenolics, except homovanillic acid and quercetin.

The results obtained agree with data published by other authors, that point out HT, tyrosol, and oleuropein (OLE) as major phenolic compounds in OP [38,39]. The obtained results show that HT was found at concentrations between 28.7 and 33.5 mg/100 g of OP, and tyrosol between 10.6–21.1 mg/100 g, with the highest concentration for extracts obtained with OH and ethanol. The OLE was only detected in hydroethanolic extracts (254.38 ± 13.24 mg/100 g for OH-assisted extraction and 369.05 ± 23.00 mg/100 g for CH extraction), as well as syringic acid (42.98 ± 3.74 mg/100 g) and apigenin (about 43 mg/100 g).

In addition to tyrosol, the extracted quantity of other compounds increased using 50% EtOH conjugated with OH; these compounds are coumaric acid (43% more concentrated compared to conventional heating for the same solvent), vanillic acid (23%), taxifolin (6%), naringenin (78%), and catechin (58%). These results may explain the higher antioxidant activity of the 50% OH-EtOH extract, previously mentioned and reported in Table 2.

Finally, it is important to mention that some compounds, such as cinnamic, syringic, and rosmarinic acids, flavonoid-rutin and stilbene-resveratrol were only extracted using OH extraction technology, not being detected in extracts obtained by CH.

Looking at the sum of individual identified phenolic compounds, OH combined with the most suitable solvent (hydroethanolic) increases the phenolic content by 24% (globally). This result can justify the increase in antioxidant activity determined by the FRAP method (15%) using the same solvent, when comparing the OH and the CH one.

Čepo et al. [38] reported for hydroethanolic (60% (*v*/*v*)) OP extracts, concentrations of approximately 51 mg/100 g for HT, 89 mg/100 g for tyrosol, and 16 mg/100 g for OLE. These authors also quantified vanillic, homovanillic, and 3,4-dihydroxybenzoic acid acids in OP extracts at concentrations between 1 to 4 mg/100 g OP. Cioffi et al. [39] reported significantly lower HT and tyrosol contents recovered from two olive cultivars (8.4–10.4 mg/kg and 20.7–21.6 mg/kg, respectively) in comparison to OLE content (81.7–83.0 mg/kg). Aliakbarian et al. [40] studied the chemical composition of OP extracts, obtained by high-pressure reactor at high temperatures and described low amounts of HT and tyrosol (21.9 and 21.1 mg/100 g dry OP, respectively) and high OLE content (203.1 mg/100 g dry OP), which are similar to our results (Table 3). The phenolic content and composition, as well as the biological properties of OP extracts may depend on variables such as olive variety, environmental conditions, geographical location, and extraction methodology. In this sense, the comparison of our results with values reported in the literature can be difficult.

Furthermore, this detailed study of the phenolic composition of OP extracts isolated with environmentally friendly and safe solvents allows us to state that these eco-extracts are rich in HT and tyrosol derivatives, phenolic acids, flavonoids, and stilbene compounds that could be used as sustainable and high added value products including new functional food, nutraceuticals, and/or therapeutic agents.

### 3.3. Structural Characterization of OP Material and OP Extracts

**Figure 1:**
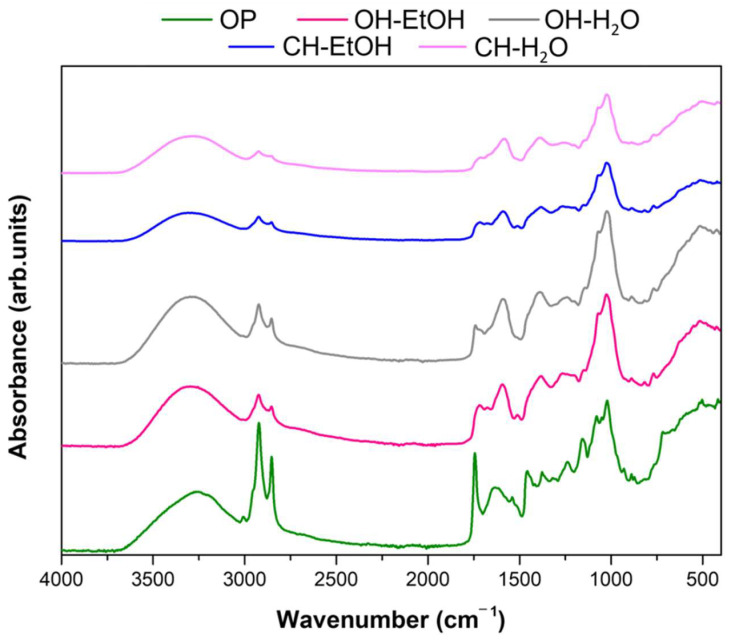
Fourier transform infrared spectra (FTIR) of the OP untreated and the extracts obtained from olive pomace (OP) waste by using ohmic heating (OH) and conventional heating (CH) techniques.

Figure 1 shows the FTIR spectra of the OP untreated, and the extracts obtained from olive pomace (OP) waste at a wavelength ranging from 400–4000 cm^–1^. ATR-FTIR technique is usually used to obtain information about the chemical groups and bonding arrangement of constituents present in the plants and plants-based extracts. The band detected at 3700–3100 cm^−1^ is attributed to molecules based on hydroxyl groups, O–H stretching vibration in phenols and aliphatic arrangements. Other representative vibration that is perceived in this region is the C–H stretch of a terminal alkyne that reveals an absorption peak at 3300 cm^−1^. The peaks at 2908 and 2852 cm^−1^ patent from C–H stretching vibrations in CH_3_–groups. Specifically, the peak at 2900, C–H stretching vibrations of asymmetric aliphatic arrangements, which may be due to the presence of less condensed structures, such as phenolics and acids. These two peaks are more intense in the extracts obtained with the OH-assisted extraction, suggesting greater extraction of compounds such as lipids (fatty acids) and hydrophilic phenolic compounds in the extracts obtained with this technology.

Absorption peaks at the wavelength between 1850–1650 cm^−1^ typically designate the existence of esther C=O groups of fatty acids.

The cellulose and lignin vibration bands were more evident at wavelengths between 1461 and 1316 cm^−1^, mainly, 1374 cm^−1^, which represents CH_2_ the bending mode of cellulose, and 1335 cm^−1^ indicates the CH distortion of ring vibration of cellulose, pectin, and polysaccharide compounds. The peaks at 1075 and 1019 cm^−1^, are typical for C-O and C-C stretching of pectins and cellulose. Pectin was identified by the absorbance between 1093 and 1650 cm^−1^ (namely, COO− asymmetric stretching of the ester group at 1630 cm^−1^, C-O stretching at 1240 cm^−1^, and O-C-O symmetric stretching of the glycosidic bond of cellulose and pectin at 1162 cm^−1^). The peaks between 1045 and 1160 cm^−1^ allowed the identification of sugars in the OP waste and its extracts. The chemical characterization of the OP extracts demonstrated the presence of phenolic compounds (Table 3), and represented in IR spectra by typical peaks at 1103 cm^−1^ and 1066 cm^−1^ can be due to an opening the structure of cyclic phenols. Small peaks between 868 to 767 cm^−1^ are typical of phenolic compounds, related to the stretching and bending vibrations of –CH of aromatic rings. Moreover, the presence of flavonoid and phenolic compounds were as also noticed due to the existence of vibrational bands allocated to the bonds O–H, C=C ring, C–OH, C–H, and C–C [41]. Though with different peak intensities, probably due to the different yields achieved, all extracts showed similar IR spectra.

The description of IR spectra results is supported by the literature, which refers to the existence of pectin, sugars, lipids, cellulose, lignin, and phenolics in OP by-products and its subsequent extracts [34,42].

### 3.4. Theoretical Absorption Percentage of Individual Phenolic Compounds (Based on Lipinski Parameters)

In silico analysis has proved to be a powerful tool to predict the behavior of those phenolic compounds identified in the OP after oral ingestion. As the human body is not capable of naturally producing phenolic compounds, they are considered as xenobiotics and follow the same pharmacokinetics pathways. The pharmacokinetic properties of these molecules can be predicted by using the Lipinski’s rule of five (LIRF) and, thus, estimate their pharmacological and physiological properties after oral ingestion. The interpretation of this rule is based on five basic physiochemical parameters that facilitates the computational prediction of the biological properties and their passive absorption during the intestinal digestion phase, as discussed in previous studies [43,44,45]. Therefore, the determination of Lipinski’s parameters in terms of the topological polar surface area (TPSA), and the theoretical percentage of absorption of the phenolic compounds from OP extracts was performed by an in-silico analysis (Table 4) and used to predict their behavior after oral intake. According to these results, the higher absorption percentages were observed for phenolic acids, with cinnamic acid being the molecule with the highest value. Among the hydroxytyrosol and tyrosol derivatives, only the HT and tyrosol molecules had a high absorption percentage, with 88.07 and 95.04%, respectively. As for the flavonoids, apigenin showed the highest absorption percentage (90.89%), followed by naringenin (78.99%), and catechin (70.92%), whereas the rest had values below 60%. According to Velderrain-Rodríguez et al. [46], theoretical absorptions above 57% have been considered as an indicator of good intestinal permeability, suggesting that rutin and hesperidin, identified in OP, are not likely being absorbed in the intestinal tract. Lastly, the stilbene resveratrol also had a high absorption percentage and no rules violations. In addition, the simulation of the Caco-2 permeability in terms of the apparent permeability coefficient (log Papp) contributes to the understanding of the biological properties of phenolic compounds after being orally ingested.

Therefore, the chemical structures of individual phenolic compounds can be used to predict their theoretical absorption percentage and can be used as an indicator of their cellular permeability. In that sense, the theoretical absorption can also be supported by its Caco-2 permeability, considering that a molecule has a high Caco-2 permeability if its P_app_ value is higher than 8 × 10^6^ cm/s. Using the pkCSM predictive model, predicted log P_app_ values > 0.90 cm/s indicates a high Caco-2 permeability of the compounds tested. In that sense, the P_app_ value observed for the phenolic compounds identified in the OP agrees with the absorption percentage previously discussed. Thus, the predicted data suggest HT, tyrosol, cinnamic acid, and *p*-coumaric and o-coumaric acids, are the HT/tyrosol derivatives and phenolic acids with higher Caco-2 permeability. As for the flavonoids, those with the higher Caco-2 permeability were apigenin, taxifolin, and naringenin. Moreover, the stilbene resveratrol also had a good permeability value. Thus, the results of this study suggest that the antiproliferative activity of OP extracts may be related to the higher absorption and permeability values of its phenolic compounds.

### 3.5. Effect of OP Extracts on Cancer Cells

Epidemiological studies in humans have shown that the consumption of phenolic compounds, and therefore also olive oil, prevent many types of cancer [47]. Therefore, plant-derived phenolic compounds could be an especially useful alternative when administered together with other drugs. Several studies have shown the anticancer properties of phenolic compounds acting through numerous signaling pathways including the death receptor (extrinsic) pathway, the mitochondrial (intrinsic) pathway, and the perforin granzyme apoptotic pathway [48,49,50].

In vitro and in vivo studies suggested that phenolic compounds from different dietary sources can play a key role to delay cancer development and progression through decreased cell proliferation, inactivation of carcinogens, angiogenesis inhibition, induction of cell cycle arrest and apoptosis and modulating immunity [51,52].

After olive oil extraction, only a low percentage of the total phenolic compounds present in the olive fruits are found in virgin olive oil. The remaining phenols (98–99%) end up in OP [7]. Among the different phenolic compounds detected in the OP, HT is one of the most important molecules due to its biological activity. Furthermore, OP also contains OLE and other relevant phenolic species. For example, like HT, OLE produces an apoptotic effect by decreasing cell viability and can also be used as an adjuvant in conventional antitumor therapies [53]. Likewise, taxifolin, also present in OP, has shown to have anticancer activity in either in vitro or in vivo models [54]. One of the most abundant flavonoids in foods of plant origin with a large number of biological properties is QUE. It is present mainly in capers, red onions, red grapes, parsley, broccoli, lovage, berries, and olive waste products [49,50]. Thus, several studies have shown that QUE has anticancer activity on different cell lines such as breast, colorectal, stomach, head and neck, lung, ovary, melanoma, and leukemia through different targets [13,14,15]. For all this, in the present work the biological properties of OP in the treatment and prevention of colon cancer are studied.

#### 3.5.1. Cell Viability Studies

**Figure 2:**
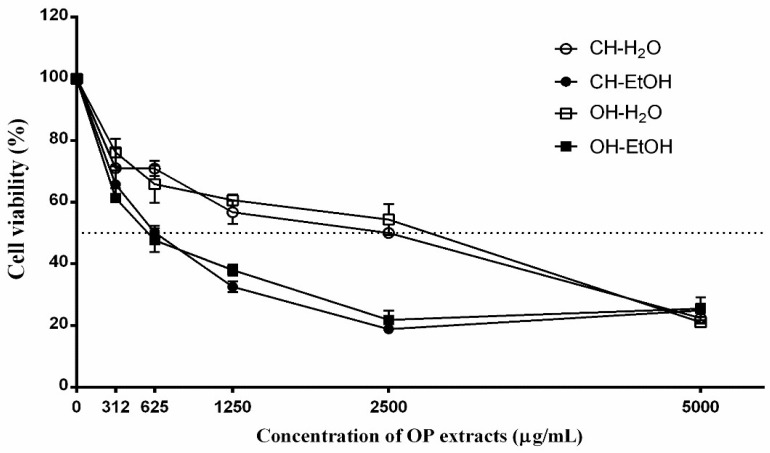
Cell viability in Caco-2 cells after incubation with olive pomace extracts (0, 312, 625, 1250, 2500, and 5000 μg/mL) for 72 h. Conventional H_2_O extract (CH-H_2_O), conventional EtOH extract (CH-EtOH), ohmic heating H_2_O extract (OH-H_2_O), and ohmic heating EtOH extract (OH-EtOH).

The effect of extracts from OP was evaluated on the viability of undifferentiated Caco-2 cells by Resaruzin assay. Initially, a range of concentrations of OP extracts (312, 625, 1250, 2500 and 5000 μg/mL) were tested (Figure 2). The conditions tested in this study were based on previous studies carried out with plant extracts [32,55]. The IC_50_ results, calculated for the Caco-2 cells treated for 72 h with tested OP extracts (CH-H_2_O, CH-EtOH, OH-H_2_O and OH-EtOH) are shown in Table 5. For future studies, the hydroethanolic extract obtained by OH-assisted extraction (OH-EtOH) was chosen as it was the one that showed the lowest IC_50_ and therefore turned out to be the most effective. This fact could be related to the greater amount of phenolic compounds found in the aforementioned extract (Table 3).

The results showed the different behavior of Caco-2 cells against the OP extracts according to whether they behaved as normal enterocytes (cells differentiated by reaching confluence) or cancerous cells (undifferentiated cells). Thus, resazurin assay revealed that the extracts can stop the uncontrolled proliferation of cancer cells without affecting normal enterocytes; so, it appears to selectively target cancer cells as shown in the IC_50_ obtained (Table 5).

#### 3.5.2. Cell Death Studies

Apoptosis is a programmed cell death, in which the externalization of phosphatidylserine alters the configuration and permeability of the cell membrane. Apoptosis can be extrinsic (death receptor) or intrinsic (mitochondrial).

**Figure 3:**
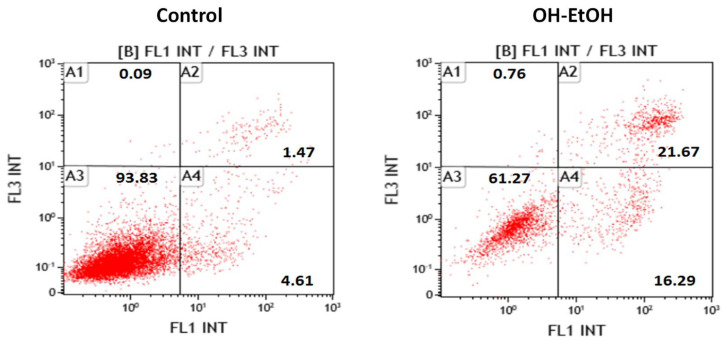
Analysis of the type of cell death induced on Caco-2 cells after 72 h incubation in Control (untreated cells) and OH-EtOH olive pomace extract at IC_50_ (692.32 μg/mL). Alive (A3), necrotic (A1), early apoptotic (A4), and late apoptotic (A2) cells are indicated in percentages.

Given that OP produced a decrease in the viability of Caco-2 cells, the next step was to determine the type of death produced using specific biomarkers by flow cytometry. The results revealed that the IC_50_ of OH-EtOH OP extract, at 72 h, induced apoptosis in the early and late stages in Caco-2 cells (Figure 3) and, thereby, reduced their ability to non-selectively react with biological targets to cause necrosis and its related side effects.

Apoptosis begins with a depolarization of the mitochondrial membrane by redistribution of hydrogen ions, altering its membrane potential that leads to the release of cytochrome c to the cytoplasm and the activation of caspase-3, which executes the apoptotic.

Since previous studies on plant extracts suggested mitochondrial dysfunction and intrinsic apoptosis induction [32,55], the mitochondrial membrane potential change and caspase 3 activity were analyzed.

**Figure 4:**
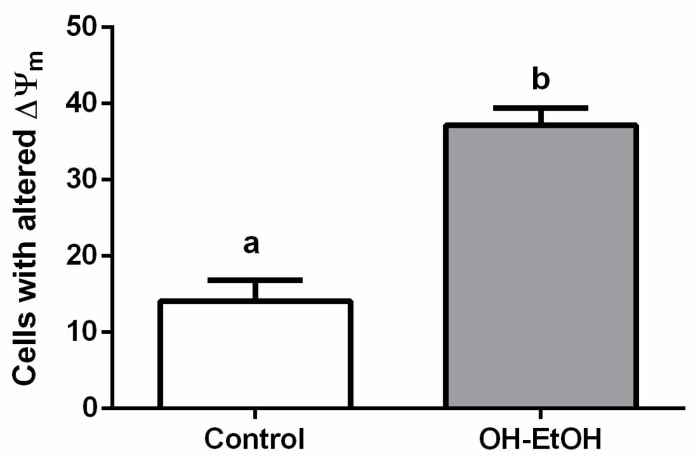
Analysis of mitochondrial membrane potential (∆Ψm) after 72 h incubation with OH-EtOH olive pomace extract at IC_50_ (692.32 μg/mL). Different letters correspond to a statistical difference (95% confidence level) between treatments.

**Figure 5:**
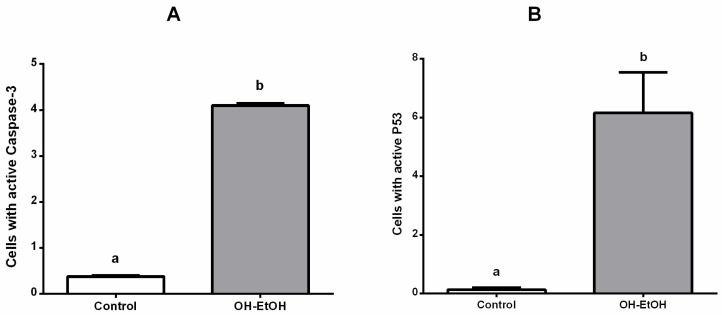
Caco-2 cells with the presence of active Caspase 3: (**A**) and P53 (**B**) after 72 h incubation with OH and OH olive pomace extract at IC_50_ (692.32 μg/mL). Different letters correspond to a statistical difference (95% confidence level) between treatments.

Flow cytometry determination showed that OP extracts significantly alter the number of cells with potential mitochondrial changes (Figure 4) and activated caspase-3 (Figure 5A). Therefore, these results support the apoptosis found by the effect of OH-EtOH extract (Figure 3).

**Figure 6:**
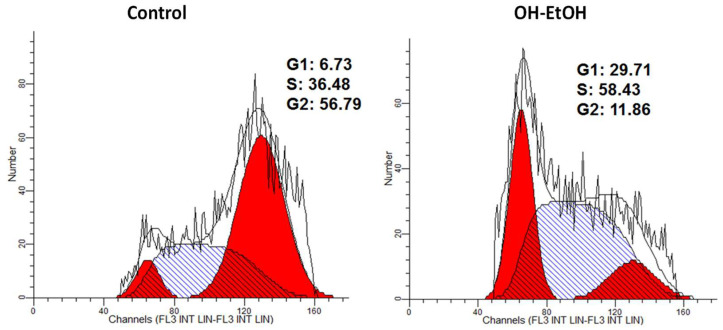
Histograms of PI stained Caco-2 cells after 72 h treatment with OH-EtOH olive pomace extract at IC_50_ (692.32 μg/mL) by flow cytometry.

Apoptosis is usually accompanied by alterations in cell cycle, as has been shown in Caco-2 cells treated with plant extracts [32,55]. Flow cytometric analysis showed that after 72 h of incubation, the OH-EtOH extract altered the cell cycle initiating arrest in G1 and S phases with a concomitant decrease in the G2 phase compared to untreated cells (Figure 6). One of the major genes that influences the regulation of the cell cycle (progression of cell division) is the tumor suppressor gene p53 [56,57]. Cancerous cells often suppress the p53 protein, upregulating anti-apoptotic Bcl 2 family proteins. Suppression of p53 also results in inhibition of caspase enzymes such as caspase 3, which is the effector gene responsible for executing apoptosis in cells [58]. Furthermore, p53 can induce apoptosis cell death in response to DNA damage [59]. The activation of p53 protein is regulated at the molecular level by several covalent modifications including acetylation, methylation, ubiquitination, and phosphorylation [60]. In this sense, it has been evidenced in DLD1 and LoVo human colon cancer cells that p53 activation reduces the constitutive activity of NF-kB probably by enhancing cytoplasmic IkB expression and contributing to apoptosis [61].

**Figure 7:**
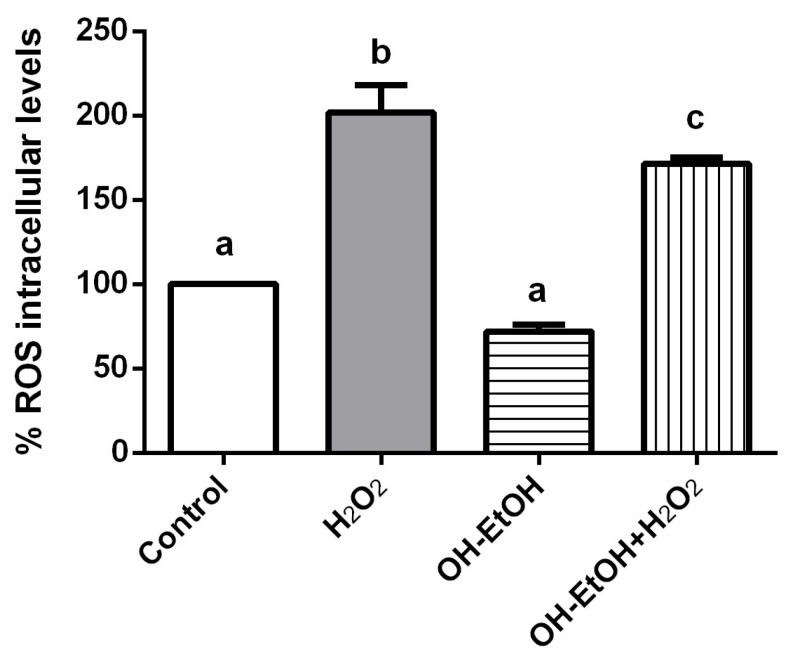
Measurements of ROS levels on undifferentiated cells in the presence or absence of H_2_O_2_ (80 mM, 20 min) after 24 h incubation with OH-EtOH olive pomace extract at IC_50_ (692.32 μg/mL). Different letters correspond to a statistical difference (95% confidence level) between treatments.

Based on all this, the active p53 was determined in cells with/without OP extract (OH-EtOH), leading to an increased amount of cells with p53 concerning the untreated cells (Figure 5B). These results correlate with those shown in Figure 4, Figure 5 and Figure 7.

Therefore, cell death or decreased viability could have been induced, at least in part, by the mitochondrial pathway with activation of p53 and caspase 3. These events cause cell cycle arrest, as p53 has been shown to greatly influence the cell cycle progression [62].

In this way, it is well known that the anticancer effect of phenolic compounds is attributed to multiple mechanisms such as induction of apoptosis, regulation of various signaling pathways, regulation of cell cycle, and activation of receptors at the level of the plasma membrane [63]. Thus, tea polyphenols upregulate wild type p53 and downregulate mutant type p53 [64]. In addition, it is documented that the overexpression of p53 protein is induced by phenolic compounds such as resveratrol, curcumin, epigallocatechin-3-gallate, quercetin, oleuropein, naringenin, and xanthohumol [65,66,67,68,69,70,71] all of which are present in our extracts (Table 3).

#### 3.5.3. Involvement of ROS in OP Caco-2 Effect

Reactive oxygen species (ROS), normally generated by the cell metabolism, are involved in a wide number of cellular functions including signaling transduction and gene transcription, and immune response. However, the overproduction of ROS is implicated in the development of diverse chronic and degenerative diseases. The interaction of ROS with cellular compounds and their function as cellular messengers to modulate diverse redox-sensitive pathways, including p53 signaling pathway or mitochondrial dysfunction, can be directly implicated in the ethology and progression of cancer [72]. The p53 protein shows both pro-oxidant and antioxidant functions in relation to the intensity of the stress, which can contribute to tumor suppression [73]. In case of mild stress, the p53 protein acts as a pro-oxidant allowing the cell to repair damage and survive. When the stress is extreme or prolonged, the p53 protein shows the antioxidant functions and subsidizes the overall process of apoptosis and/or autophagy [74].

Natural products targeting ROS-modulated pathways may be a promising tool for cancer therapy. The ethanolic extract from *Salix aegyptiaca* inhibited the colorectal carcinoma (HCT-116) cells proliferation, induced cell apoptosis, and augmented the stability of the wild type p53 protein; however, the mutant p53 protein in other colorectal cancer (HT-29) cells was diminished in a process related to a decreased superoxide anion production [75]. In another study, (+)-cyanidan-3-ol significantly reduced hepatic cancer (HepG2) cell proliferation and tumor growth in mice with a mechanism associated with a reduced ROS production, up-regulation of p53 protein and BAX, and down-regulation of bcl-2, activator protein 1 (AP-1), and nuclear factor NF-kB [76].

Several studies have shown the antioxidant power of the different parts of the *Olea europaea* plant as well as of the olive processing by-products evaluated by DPPH and ABTS [77,78,79,80]. The obtained results in the present work with OP extracts, also present an antioxidant effect as reflected in Table 2.

In addition, to investigate whether ROS was implicated in the olive pomade-induced anti-proliferation effect, ROS levels in the cells were determined based on the reaction between ROS and DCFH-DA. The assays were carried out by treating the cells with OH-EtOH from OP in the presence or absence of H_2_O_2_. Hydrogen peroxide is a compound used to simulate a pro-oxidative environment characteristic of degenerative diseases such as cancer in 2D cell cultures. Results indicated OH-EtOH reduces cellular levels of ROS in Caco-2 cells (Figure 7). Therefore, this event could also induce G1/S phase arrest and apoptosis via ROS-mediated mitochondrial dysfunction in which process the p53 protein would be involved.

### 3.6. Antioxidant Capacity of Olive Pomace Extracts on a Model Intestinal Barrier

Chronic oxidative stress on the mucosal barrier is strongly correlated with gastrointestinal tract malignancies such as Crohn’s, inflammatory bowel disease (IBD), or colorectal cancer [81,82]. The sources of pro-oxidant agents are wide, from drugs and compounds present in food to intestinal pathogens [83,84]. In this context, dietary intervention with antioxidant supplementation has been suggested as a novel therapeutic approach to reduce oxidative stress-related damage of the intestinal barrier. The antioxidant capacity of plant extracts is linked to their clinical application on gastrointestinal diseases related to oxidative stress. Thus, Catanzaro et al. [85] found that incubation of differentiated Caco-2 cells with *Boswellia serrata* extracts protected against H_2_O_2_-induced damage and maintained monolayer integrity by avoiding the disassembly of tight junctions. This cell line spontaneously acquires the phenotypic features of non-cancerous enterocytes after reaching confluence (differentiated cells). Monolayer Caco-2 cells form tight junctions and present the cylindrical polarized morphology of enterocytes, expressing functional microvilli on the apical membrane [86]. Therefore, differentiated Caco-2 cells have been established as an acceptable in vitro intestinal barrier model [87].

Some research has shown that olive phenolic compounds possess biological properties and probably play a role in the prevention of various diseases such as inflammatory bowel disease [8]. In the present study, an extract of olive pomace (OH-EtOH) was tested as a supplement on the human intestinal cell in culture (Caco-2).

**Figure 8:**
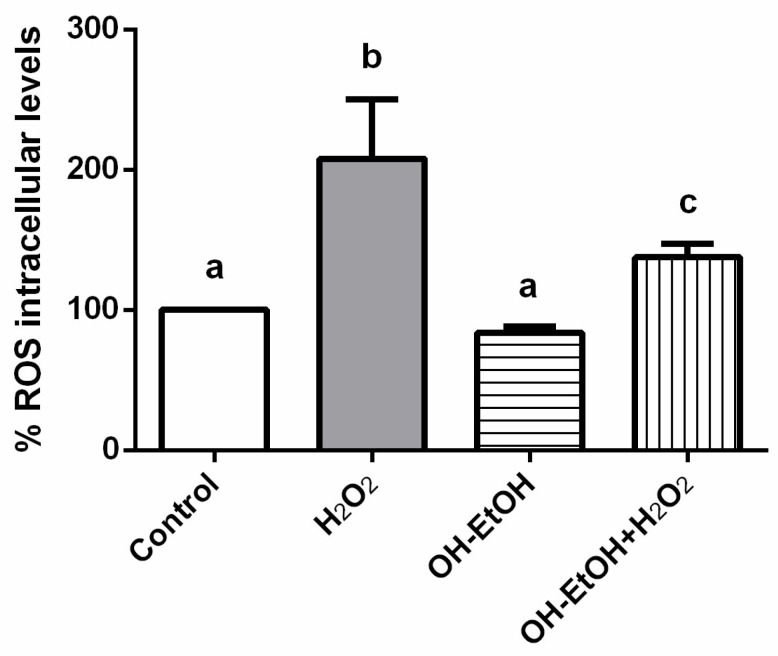
Measurements of ROS levels on Caco-2 differentiated cells in the presence or absence of H_2_O_2_ (80 mM, 20 min) with OH-EtOH olive pomace extract at IC_50_ (692.32 μg/mL) after 24 h incubation. Different letters correspond to a statistical difference (95% confidence level) between treatments.

Given the high antioxidant capacity found in the OH-EtOH extracts measured by the DPPH and FRAP assays (Table 2), and the decrease in ROS produced by these extracts in cancer cells (Figure 7), it seemed interesting to test the same concentration of OP (IC_50_) on an intestinal barrier model (Caco-2 differentiated), in the presence or absence of H_2_O_2_. The results obtained with olive pomace extracts showed an antioxidant effect in Caco-2 cells (Figure 8) and, therefore, they could have a potential application in the management of gastrointestinal diseases related to oxidative stress.

These results confirm the therapeutic potential of phenolic compounds, and specifically of olive pomace in intestinal stress diseases. Although intervention studies are necessary to confirm the clinical significance of our results, the results found here can be taken into account in the exploitation of olive pomace as a formulation of new foods with added value.

## 4. Conclusions

In the present work, we focus our attention on the use of the phenolic compounds present in residues, such as the OP, obtained from the processing of olive fruit to obtain oil. It has been shown that hydroethanolic solvents were the best for extracting the phenolic fraction (as expected) and that hydroethanolic solvents in combination with OH allowed the achievement of an even higher yield in these compounds. The extracts have been tested on both undifferentiated and differentiated Caco-2 cells, used as models of colon cancer cells and normal enterocytes (healthy intestinal cells), respectively. The OH-EtOH extracts have shown the highest antiproliferative effect on colon cancer cells, probably due to its higher content of phenolic compounds. These extracts stopped the cell cycle in the G1/S phase, showing a significant increase in the activity of the p53 protein. Likewise, death by apoptosis was determined with changes in the mitochondrial membrane potential and activation of caspase 3, which shows an intrinsic apoptosis with alteration of redox balance (decrease in intracellular levels of ROS). On the intestinal barrier, OP extracts showed an antioxidant effect, which makes them interesting in the prevention of intestinal diseases related to oxidative stress, such as intestinal cancer. In silico studies have shown that tyrosol, cinnamic acid, and apigenin are the phenolic compounds with the highest theoretical absorption and Caco-2 permeability compared to the other species found in the OP extracts. Thus, it is quite probable that these molecules are responsible for that intracellular decrease in ROS. In vitro experiments represent the first step in evaluating the health effect of new functional ingredients. Therefore, the bioactive principles present in olive pomace can be recovered by applying OH as green technology and used for food, nutraceutical, and biomedical applications, reducing environmental waste, and facilitating the circular economy. Although, future clinical trials are necessary to advance the use of these extracts for designing functional foods.

## Figures and Tables

**Table 1:** Chemical composition of olive pomace.

Proximate Composition (g/100 g Dry OP)		Mineral Element (mg/Kg Dry OP)	
Cellulose ^a^	16.16 ± 0.78	Potassium	2798.44 ± 29.64
Hemicellulose	18.96 ± 1.79	Calcium	352.03 ± 23.01
Xylose	15.32 ± 0.45	Magnesium	145.41 ± 16.54
Arabinose	3.64 ± 0.20	Iron	12.24 ± 2.71
Acetyl group	3.75 ± 0.73	Sodium	16. 42 ± 0.67
Lignin	31.69 ± 1.40	Aluminum	16.93 ± 2.08
Insoluble	20.47 ± 1.31	Manganese	1.56 ± 0.22
Soluble	11.22 ± 0.13	Zinc	2.92 ± 0.36
Protein	5.66 ± 0.31	Copper	2.73 ± 0.29
Fat	12.06 ± 0.79	Boron	4.02 ± 0.52
Ashes	4.55 ± 0.22	Barium	0.18 ± 0.03
Total extractives ^b^	25.23 ± 1.88		

^a^ Glucose content; ^b^ Water and ethanol extractives. Results are expressed as mean ± SD (*n* = 3).

**Table 2:** Total phenolic content (TPC), antioxidant activity (DPPH and FRAP assay) and extraction yield of the aqueous and hydroethanolic olive pomace extracts obtained by ohmic heating (OH) and conventional heating (CH) techniques.

Method	Solvent	TPC (mg GAE/g OP)	Antioxidant Activity		Yield (%)
			DPPH * (µmol TE/g OP)	FRAP (µmol Fe(II)/g OP)	
OH	H_2_O	12.08 ± 1.52 ^a^	3.23 ± 0.10 ^b^	80.41 ± 1.12 ^a^	28.54 ± 0.14 ^a^
	EtOH 50%	17.67 ± 3.12 ^b^	3.82 ± 0.04 ^a^	150.16 ± 9.29 ^b^	27.39 ± 1.54 ^ac^
CH	H_2_O	12.24 ± 0.88 ^a^	3.36 ± 0.03 ^b^	80.45 ± 2.45 ^a^	24.70 ± 0.27 ^b^
	EtOH 50%	16.89 ± 0.76 ^b^	3.56 ± 0.05 ^c^	130.34 ± 2.39 ^c^	25.60 ± 0.19 ^bc^

* DPPH inhibition concentration at 50% (IC_50_). Values are expressed as mean ± SD (*n* = 3). Different letters in the same column correspond to statistically different between samples for a 95% confidence level.

**Table 3:** Identification and quantification of individual phenolic compounds in the aqueous and hydroethanolic olive pomace extracts obtained by ohmic heating (OH) and conventional heating (CH) techniques.

Phenolic Compound	OH		CH	
	H_2_O	EtOH 50%	H_2_O	EtOH 50%
Hydroxytyrosol and tyrosol derivatives				
Hydroxytyrosol	28.71 ± 0.42 ^a^	33.36 ± 0.57 ^b^	31.20 ± 0.29 ^c^	33.49 ± 0.40 ^b^
Tyrosol	10.63 ± 0.06 ^a^	21.09 ± 0.12 ^b^	11.13 ± 0.15 ^a^	11.09 ± 0.99 ^a^
Oleuropein	n.d.	254.38 ± 13.24 ^a^	n.d.	369.05 ± 23.00 ^b^
Phenolic acids				
Caffeic acid	n.d.	2.59 ± 0.47 ^a^	n.d.	2.27 ± 0.35 ^a^
Cinnamic acid	18.45 ± 0.95 ^a^	19.99 ± 0.46 ^b^	n.d.	n.d.
*p*-Coumaric acid	44.99 ± 3.01 ^a^	48.41 ± 2.84 ^a^	20.91 ± 0.91 ^b^	48.76 ± 1.49 ^a^
*o*-Coumaric acid	23.26 ± 1.24 ^a^	69.81 ± 1.14 ^b^	25.45 ± 0.41 ^a^	48.93 ± 1.02 ^c^
Ferulic acid	22.31 ± 1.38 ^a^	13.83 ± 1.28 ^b^	23.60 ± 1.22 ^a^	22.40 ± 0.51 ^a^
Vanillic acid	39.51 ± 0.48 ^a^	72.77 ± 1.11 ^b^	43.96 ± 1.79 ^c^	59.41 ± 0.50 ^d^
3,4-Dihidroxibenzoic acid	14.20 ± 0.28 ^a^	17.61 ± 0.94 ^b^	15.60 ± 0.37 ^ac^	16.61 ± 0.39 ^bc^
Syringic acid	n.d.	42.98 ± 3.74 ^a^	n.d.	n.d.
Ellagic acid	142.93 ± 4.49 ^a^	147.82 ± 3.40 ^a^	162.01 ± 0.61 ^b^	148.40 ± 3.85 ^a^
Homovanillic acid	103.45 ± 6.24 ^a^	75.54 ± 0.56 ^b^	118.62 ± 5.56 ^c^	n.d.
Rosmarinic acid	22.05 ± 0.80 ^a^	62.79 ± 2.27 ^b^	n.d.	n.d.
Flavonoids				
Apigenin	n.d.	43.13 ± 1.28 ^a^	n.d.	42.48 ± 1.31 ^a^
Rutin	21.33 ± 0.62 ^a^	31.46 ± 2.68 ^b^	n.d.	n.d.
Taxifolin	83.10 ± 2.23 ^a^	94.44 ± 4.01 ^b^	85.82 ± 3.40 ^a^	89.03 ± 2.42 ^a^
Naringenin	125.11 ± 0.56 ^a^	247.42 ± 10.19 ^b^	99.96 ± 4.62 ^c^	137.67 ± 10.14 ^a^
Hesperidin	137.30 ± 5.22 ^a^	93.15 ± 4.24 ^b^	62.77 ± 1.46 ^c^	85.75 ± 2.44 ^b^
Quercetin	168.62 ± 15.47 ^a^	134.94 ± 0.89 ^b^	206.01 ± 6.29 ^c^	134.14 ± 2.85 ^b^
Catechin	63.15 ± 2.39 ^a^	77.68 ± 1.06 ^b^	55.29 ± 1.58 ^c^	49.04 ± 0.78 ^d^
Stilbene				
Resveratrol	6.68 ± 1.02 ^a^	10.34 ± 0.29 ^b^	n.d.	n.d.
Total	1075.88	1615.54	962.23	1298.53

All the results are expressed as mean ± SD (*n* = 3) and reported as milligrams of phenolic compound per 100 g of dry material (mg/100 g OP). Different letters in the same row correspond to a statistical difference (95% confidence level) between the samples with respect to analyzed compound; n.d.: not detected.

**Table 4:** Theoretical absorption percentage based on Lipinski parameters of OP phenolic compounds.

Phenolic Compound	MW	TPSA	Log P	No. atoms	Hydrogen Bonds Acceptors	Hydrogen Bonds Donors	Rotatable Bonds	Molecular Volume (Å3)	Violations to LIRF	% ABS	Log Papp
Hydroxytyrosol and tyrosol derivatives											
Hydroxytyrosol	154.16	60.68	0.52	11	3	3	2	141.70	0	88.07	1.09
Tyrosol	138.17	40.46	1.00	10	2	2	2	133.68	0	95.04	1.69
Oleuropein	540.52	201.68	−0.36	38	13	6	11	466.31	3	39.42	0.06
Phenolic acids											
Caffeic acid	180.16	77.75	0.94	13	4	3	2	154.50	0	82.18	0.63
Cinnamic acid	148.16	37.30	1.91	11	2	1	2	138.46	0	96,13	1.71
*p*-Coumaric acid	164.16	57.53	1.43	12	3	2	2	146.48	0	89.15	1.21
o-coumaric acid	164.16	57.53	1.67	12	3	2	2	146.48	0	89.15	1.21
Ferulic acid	194.19	66.76	1.25	14	4	2	3	172.03	0	85.97	0.17
Vanillic acid	168.15	66.76	1.19	12	4	2	2	144.61	0	85.97	0.33
3,4-Dihidroxibenzoic acid	154.12	77.75	0.88	11	4	3	1	127.08	0	82.17	0.49
Syringic acid	198.17	76	1.20	14	5	2	3	170.15	0	82.78	0.49
Ellagic acid	302.19	141.33	0.94	22	8	4	0	221.78	0	60.24	0.33
Homovanillic acid	182.18	66.76	0.70	13	4	2	3	161.41	0	85.96	0.26
Rosmarinic acid	360.32	144.52	1.63	26	8	5	7	303.54	0	59.14	−0.93
Flavonoids											
Apigenin	270.24	90.89	2.46	20	5	3	1	224.05	0	90.89	1.00
Rutin	610.52	269.43	−1.06	43	16	10	6	496.07	3	16.04	−0.94
Taxifolin	304.25	127.44	0.71	22	7	5	1	246.32	0	65.03	0.92
Naringenin	272.26	86.99	2.12	20	5	3	1	230.26	0	78.99	1.02
Hesperidin	610.57	234.30	−0.55	43	15	8	7	511.79	3	28.16	0.50
Quercetin	302.24	131.35	1.68	22	11	7	1	240.08	0	63.68	−0.22
Catechin	290.27	110.37	1.37	21	6	5	1	244.14	0	70.92	−0.28
Stilben											
Resveratrol	228.25	60.68	2.99	17	3	3	2	206.92	0	88.06	1.17

MW = Molecular weight; TPSA = total polar surface area; Log *p* = octanol–water partition coefficient; Violations to LIRF = Violations to Lipinski’s rule of five; % ABS = Theoretical absorption percentage; log Papp = logarithm of the apparent permeability coefficient.

**Table 5:** IC50 (μg/mL) values of olive pomace extracts on Caco-2 undifferentiated and differentiated cells upon 72 h incubation.

Extract	Caco-2 Undifferentiated	Caco-2 Differentiated
CH-H_2_O	2256.1 ± 237.56 ^a^	>5000 ^a^
CH-EtOH	747.99 ± 140.97 ^b^	>5000 ^a^
OH-H_2_O	2817.10 ± 53.28 ^a^	>5000 ^a^
OH-EtOH	692.32 ± 63.58 ^b^	4026 ± 274 ^b^

Different letters in the same column correspond to a statistical difference (95% confidence level) between treatment.

## Data Availability

Data is contained within the article.
